# The complete chloroplast genome sequence of *Dictamnus albus L.*

**DOI:** 10.1080/23802359.2022.2081097

**Published:** 2022-06-14

**Authors:** Jiahan Liu, Zhen Wang, Weichao Ren, Chi Liu, XiangQuan Li, Jiayong Han, Wei Ma

**Affiliations:** a Pharmacy College, Heilongjiang University of Chinese Medicine, Harbin, China; b Faculty of Electrical Engineering and Information Technology, Technical University of Chemnitz, Chemnitz, Germany; c Yichun Branch of Heilongjiang Academy of Forestry, Yichun, China; d Jiangsu Kanion Pharmaceutical Co. Ltd., Lianyungang, China; e State Key Laboratory of New-tech for Chinese Medicine Pharmaceutical Process, Lianyungang, China

**Keywords:** *Dictamnus albus*, complete chloroplast genome, phylogeny, Rutaceae

## Abstract

*Dictamnus albus L.* refers to a perennial herb with both ornamental and medicinal value. In the present study, we obtained the complete chloroplast genome sequence of *D. albus* through high-throughput sequencing. The length of the chloroplast genome was 157,139 bp, while the large single-copy and small single-copy regions were 84,478 bp and 18,587 bp, respectively. The pair of inverted repeat sequences was 27,037 bp, and the GC content was 38.5%. A total of 132 genes were annotated, including 87 protein-coding genes (PCGs), eight ribosomal RNA (rRNA) genes, and 37 transfer RNA (tRNA) genes. The chloroplast genomes of *D. albus* and eight species of Rutaceae were subjected to maximum-likelihood phylogenetic tree analysis. *D. albus* was found to be most closely related to *Orixa japonica*.

*Dictamnus albus L.* 1753 is a perennial herb belonging to the genus *Dictamnus* of the Rutaceae family. Its main production area is in northern China. *D. albus* is typically 30–90 cm tall, with a neat and compact plant shape. The whole plant has a special fragrance, and the feathery compound leaves have a lemon-like smell and are green and shiny with a certain ornamental value (Wang 2021). The root bark of *D. albus* can be used to clear away heat and dampness as well as expel wind and detoxify (Jia et al. 2018). Modern research has demonstrated that white flesh not only has medicinal value but also has health care and beauty-related effects (Wang et al. 2013). The chloroplast genome of plants has the advantages of a conserved structure and easy sequencing with wide application in studies on the systematic evolution of various plant groups (Daniell et al. 2016). The chloroplast genome of *D. albus* provides more detailed and complete data for research on Rutaceae plants.

The samples of *D. albus* were collected from Yichun City, Heilongjiang Province, China, on 10 June 2020 (N 45°72′59″, E 126°65′16″). Voucher specimens were deposited at the Department of Traditional Chinese Medicine Resources, Heilongjiang University of Traditional Chinese Medicine (Dr. Ren, lzyrenweichao@126.com) (registration number: HRB20210610001), whereas the DNA samples were stored at the Molecular Laboratory of Heilongjiang University of Traditional Chinese Medicine (Harbin, China). The DNA of fresh leaves of *D. albus* was extracted by employing the CTAB method and sequenced by Illumina NovaSeq (Benagen Co., Wuhan, China). A total of 5.68 G of raw data were obtained, and the average fragment size range was 150 bp. GetOrganelle V1.7.5 software was used for chloroplast genome splicing (Jin et al. 2020). GeSeq software was employed for functional annotation (Tillich et al. 2017). Assembly annotation using Rutaceae plant *Ruta graveolens* was reference genome. Finally, MEGA X software was adopted to construct a maximum-likelihood (ML) tree. The whole genome sequence of *D. albus* was submitted to GenBank with the accession number MZ750957.

The chloroplast genome of *D. albus* is composed of a quadripartite structure with a total size of 157,139 bp covering a large single-copy (LSC) region of 84,478 bp, a small single-copy (SCC) region of 18,587 bp, and two inverted repeat (IR) regions of 27,037 bp each. The total nucleotide composition was 30.4% A, 31.1% T, 19.6% C, and 18.9% G. In total, 132 genes were annotated, including 87 protein-coding genes (PCGs), 37 transfer RNA (tRNA) genes, and eight ribosomal RNA (rRNA) genes, which is consistent with most Rutaceae plants.

The chloroplast genomes of 10 plants downloaded from the NCBI database were compared; nine were Rutaceae plants, including an early *Dictamnus dasycarpus* genome sequence. In addition, *Arabidopsis thaliana* was used as the outgroup. MAFFT was employed for performing sequence analysis (Rozewicki et al. 2019). The phylogenetic relationship of *D. albus* was constructed based on the ML method with 1000 bootstrap replications in MEGA X software (Kumar et al. 2018) (Figure 1). According to these results, the *D. albus* sequence was clustered on the same branch as the Rutaceae chloroplast genome sequences from the NCBI database, as expected. Compared with the early *D. albus* chloroplast sequence, a pair of genes, including *ycf15*, with unknown functions were annotated in the IRS region, which further improved the quality of the chloroplast genome database. Based on the phylogenetic results, we concluded that *D. albus* is most closely related to *Orixa japonica*. The chloroplast genome data of *D. albus* provide more detailed and complete information for research on *Dictamnus*, thus laying a foundation for plant identification and the analysis of genetic evolution.

**Figure 1.**
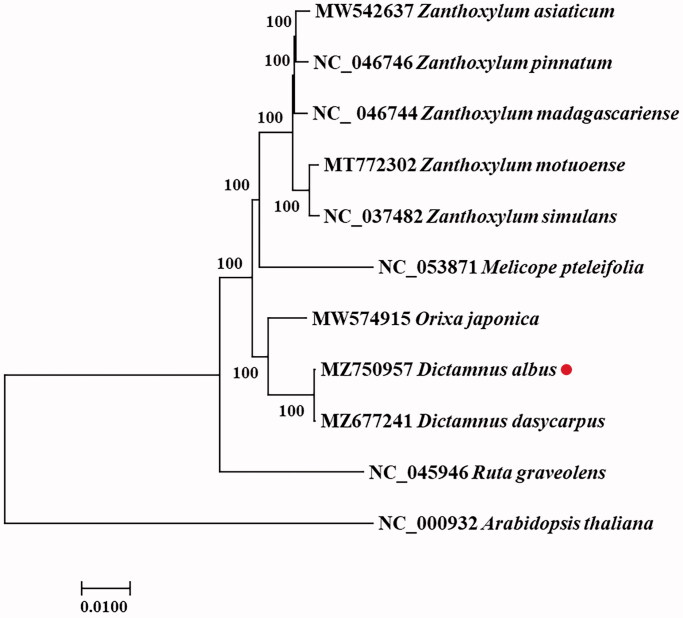
Phylogenetic tree reconstruction of 10 samples using maximum-likelihood based on complete chloroplast genome. Sequence data were obtained from NCBI database; represents the species in this study.

## Data Availability

The genome sequence data that support the findings of this study are openly available in GenBank of NCBI at https://www.ncbi.nlm.nih.gov/ under the accession no. MZ750957. The associated BioProject, SRA, and Bio-Sample numbers are PRJNA763242, SRR15900239, and SAMN21436696, respectively.
